# Challenges for anaesthesia for robotic-assisted surgery in the elderly: A narrative review

**DOI:** 10.1097/EA9.0000000000000019

**Published:** 2023-02-09

**Authors:** Paola Aceto, Claudia Galletta, Chiara Cambise, Giovanni Punzo, Ersilia Luca, Chiara Schipa, Liliana Sollazzi

**Affiliations:** From the Dipartimento di Scienze dell’ Emergenza, Anestesiologiche e della Rianimazione, Fondazione Policlinico Universitario A. Gemelli IRCCS, Rome, Italy (AP, CG, CC, GP, EL, CS, LS), Dipartimento di Scienze biotecnologiche di base, cliniche intensivologiche e perioperatorie, Università, Cattolica del Sacro Cuore, Rome, Italy (AP, LS)

## Abstract

Steep and reverse Trendelenburg positions are often used in robotic-assisted surgery (RAS) to improve surgical access. The elderly are particularly vulnerable to the cardiovascular effect of the combination of pneumoperitoneum and these extreme positions. Falls in both cardiac output (CO) and mean arterial pressure (MAP) caused by pneumoperitoneum are enhanced in reverse Trendelenburg. Hypotension with dangerous cerebral and myocardial hypoperfusion may occur. Caution should be exercised in patients with low cardiac reserve and the degree of peri-operative risk should dictate the level of haemodynamic monitoring employed. The effects of pneumoperitoneum on CO are less pronounced in the standard Trendelenburg position due to gravity, but head-down combined with pneumoperitoneum can increase both MAP and systemic cardiovascular resistance. However, in patients with impaired myocardial contractility, the head-down position may lead to cardiac failure. In addition, the adverse respiratory effects of pneumoperitoneum, which include reduction of pulmonary compliance and functional residual capacity, may be exacerbated by steep Trendelenburg. At the same time, hypercarbia resulting from CO_2_ insufflation can lead to an increase in stasis of brain blood flow and intracranial pressure with possible repercussions on cognitive functions in the elderly. Another problem is the increase in intra-ocular pressure during steep Trendelenburg, and injury to the optic nerve has been reported after robot-assisted prostatectomy. Finally, strategies to use the lowest possible pneumoperitoneum pressure are considered to reduce possible complications. Moreover, the extreme positions should be limited only to the time strictly necessary for surgery and should be avoided in high-risk patients.

**KEY POINTS**

- Robotic-assisted surgery can be safely performed in the elderly population, even if caution should be used in case of concomitant diseases.
- Strategies to use the lowest possible pneumoperitoneum pressure are considered to reduce possible complications.
- The extreme positions should be limited only to the time strictly necessary for surgery and should be avoided in high-risk patients.

## Introduction

There is consistent evidence that robotic-assisted surgery (RAS) rather than traditional laparotomy improves outcomes without affecting safety in the growing elderly population.^1^ The main benefits are a decreased complication rate, reduced blood loss and shorter hospital stay. According to Lavoue *et al.*^2^, the overall complication rate for adverse events is 21% in RAS versus 66% with the open approach. Extreme positions that include steep Trendelenburg for uro-gynecological surgery, and reverse Trendelenburg for abdominal surgery^4^ are used to facilitate surgical access. Concerns associated with prolonged pneumoperitoneum combined with extreme positioning make assessment of each individual's comorbidities, particularly severe cardiopulmonary disease, intracranial pathology, and advanced glaucoma, essential^3^ if adverse effects on the respiratory, cardiovascular and cerebral systems are to be avoided without losing sight of the potential advantages of RAS.

It is important to remember that once the patient is docked to the robot, the position cannot be reversed without undocking. Therefore, every surgical team must have an emergency plan ready if timely undocking of the robot is needed.^5^

This review considers the cardiovascular, respiratory, ocular and cerebral physiopathology during RAS and identifies strategies to address specific issues that may arise in an elderly patient.

## Haemodynamic effects of pneumoperitoneum

Pneumoperitoneum is responsible for increased intra-abdominal pressure leading to neuro-endocrine vasoactive compounds such as catecholamines and vasopressin, being released in response to gas insufflation. This brings about an increase in systemic vascular resistance (SVR) and a subsequent decrease in preload and reduced inotropism: the latter is further exacerbated by inferior vena cava compression. Reduction in cardiac filling pressures results in decreased stroke volume (SV), which may lead to a decrease in mean arterial pressure. In patients with impaired myocardial function, the increase in cardiac work and myocardial oxygen consumption may lead to serious cardiac complications.^6^

In RAS, there is also a risk of cardiac arrhythmias, such as severe bradycardia due to vagally mediated cardiovascular reflexes initiated by rapid peritoneum distension due to carbon dioxide (CO_2_) insufflation. Limiting intra-abdominal pressure (IAP) to <8–10 mmHg and using a low flow rate for CO_2_ insufflation are effective in preventing pathophysiological changes during pneumoperitoneum.^7^ Pneumoperitoneum may also have an impact on splanchnic perfusion which may be greatly impaired in the elderly. Increased intra-abdominal pressure triggers a reduction in hepatic and renal blood flow, which in turn changes the pharmacokinetics of drugs metabolised by these organs. Therefore, the doses of potentially nephrotoxic or hepatotoxic drugs should be reduced.^8^

## Haemodynamic effects of pneumoperitoneum combined with reverse Trendelenburg position

The reduction of both cardiac output (CO) and mean arterial pressure (MAP) caused by pneumoperitoneum is enhanced in the reverse Trendelenburg position. Hypotension with dangerous cerebral and myocardial hypoperfusion may occur. Caution should be used in patients with low cardiac reserve and the level of intra-operative haemodynamic monitoring should be adjusted according to the degree of peri-operative risk posed by the patient.^4^ In major abdominal RAS, the availability of transoesophageal echocardiography is recommended in the event of peri-operative haemodynamic instability.^9^

RAS often has a long duration, so it is important to avoid fluid imbalance. Goal-directed fluid management using dynamic indices, including stroke volume and stroke volume variation, and pulse pressure variation, could help maintain an optimum intravascular volume.^4^ In the absence of complications from bleeding, a zero fluid balance should be followed as it could help avoid hypervolaemia, dilutional anaemia and reduce the risk of head and neck oedema.^10^ If hypotension occurs, the use of a vasopressor should be considered to avoid excessive fluid infusion.^9^

## Haemodynamic effects of pneumoperitoneum combined with the Trendelenburg position

The effects of pneumoperitoneum on CO are less pronounced in the head-down position because of gravitational effects, but head-down combined with pneumoperitoneum can increase both mean arterial pressure and systemic cardiovascular resistance, as demonstrated by Falabella *et al.*^11^ using transoesophageal echocardiography in robotic-assisted laparoscopic prostatectomy (RALP). These changes are due to increased intra-abdominal pressure compressing the aorta and increasing afterload. Lestar *et al.*^12^ and Danic *et al.*^13^ showed an increase in MAP by 25% and 17%, respectively. However, in patients with impaired myocardial contractility, the head-down position may precipitate cardiac failure. Lestar *et al.*^12^ showed that right and left ventricular stroke work indices increased by 65% and 35%, respectively. The increase in myocardial oxygen consumption can lead to different cardiac complications according to whether there is impaired myocardial function or ischaemic cardiac disease. Head-down may also increase central venous pressure, and pulmonary arterial and wedge pressure. Patients suffering from severe pulmonary hypertension are not ideal candidates for RAS in which steep Trendelenburg is required.^6^ However, to our knowledge, there is not a threshold in pulmonary artery pressure that does not allow the use of RAS.

Steep Trendelenburg (≥30°), when needed, should be limited only for the time strictly necessary for surgery and should be avoided in high risk patients.^4^ The extraperitoneal approach should be considered when possible, in order to reduce the Trendelenburg angle and/or the CO_2_ insufflation pressure.^14^

## Pulmonary implications

Pneumoperitoneum is known to stiffen the chest wall and respiratory system.^3^ Lestar *et al.* reported an important reduction in lung compliance from 60 ml cmH_2_O^−1^ to 28 ml cmH_2_O^−1^ during RALP. Similar results were found by Kalmar *et al.*^15^ This reduction results in increased inspiratory pressures with risk of barotrauma.^12,15^

The pulmonary implications of increased intrathoracic pressure caused by pneumoperitoneum include a decrease in pulmonary compliance and functional residual capacity (FRC) with a potential for atelectasis and ventilation/perfusion mismatch. Atelectasis and ventilation/perfusion mismatch can induce hypoxaemia.^7^ These changes are more pronounced in the head-down position due to cephalad displacement of the diaphragm.

A protective ventilation strategy based on low tidal volume associated with positive end-expiratory pressure (PEEP) is recommended to increase the FRC and improve respiratory mechanics.^3^ Alveolar recruitment manoeuvres may improve oxygenation. This approach minimises stress and strain and reduces postoperative respiratory complications.^4^ PEEP improves dynamic compliance, with beneficial effects on damage from alveolar opening and closing, and reduces the incidence of atelectasis. However, in the elderly, the beneficial effects of applying PEEP should be balanced against its potential impairment of cardiac output.^13,15^

Several single-centre studies have demonstrated the superiority of pressure-controlled versus volume-controlled ventilation as it allows lower peak pressures and improves the ratio of arterial oxygen partial pressure to fractional inspired oxygen.^16,17^

Systemic CO_2_ absorption must be considered in the anaesthetic management. Mechanical ventilation should be adjusted to avoid the hypertension and dysrhythmias which may occur as a result of hypercarbia. Reabsorption of CO_2_, when combined with difficult ventilation and increased dead space, can lead to respiratory acidosis.^4^

Elderly patients undergoing RAS are often affected by chronic obstructive pulmonary disease (COPD). Intra-operative and postoperative complications are more common in these patients and can lead to prolonged hospital stay and mortality.^4^ In high risk patients, including those with COPD, the use of high flow nasal cannula or noninvasive ventilation in the peri-operative period may improve respiratory status and, consequently, may reduce the risk of atelectasis and re-intubation.^3^

The use of deep neuromuscular block (dNMB) may help to avoid the use of high insufflation pressure preventing the related haemodynamic and respiratory complications.^3^ However, the use of dNMB requires both neuromuscular monitoring and adequate reversal, by direct or indirect antagonists, to prevent postoperative residual curarisation, given that that the elderly are more susceptible to adverse postoperative respiratory events.^18^

## Changes in intracranial pressure and postoperative cognitive disorders

The combination of pneumoperitoneum and steep Trendelenburg can increase intracranial pressure (ICP), even if rarely, at values >20 mmHg; however, in patients without neurological disorders, this effect has no clinical impact. Kamine *et al.*^19^, in a small number of patients (*n* = 9) undergoing laparoscopy and needing ventriculoperitoneal shunt insertion, demonstrated that the ICP increased linearly with intra-abdominal pressure up to 25 mmHg. However, no neurological complications were found. Robba *et al.* on the other hand, recommend care during laparoscopy in patients at risk of developing intracranial hypertension, such as those with cerebral tumours or co-existing neurological disease. According to Robba *et al.*^20^, noninvasive assessments of ICP, including ultrasonic measurement of optic nerve sheath diameter (ONSD) and transcranial Doppler (TCD) techniques, could be useful to assess the risk and eventually treat pathological increases of intracranial pressure with osmotic agents, hyperventilation or conversion to an open procedure. They have demonstrated that the optic nerve sheath diameter, the diastolic component of the TCD cerebral blood flow velocity, and pulsatility index all increased significantly after the combination of pneumoperitoneum and head-down positioning.^21,22^ According to Kim *et al.*^23^ in 15% of patients, ONSD increased by values equivalent to an ICP >20 mmHg during pneumoperitoneum combined with the head down position, but Verdonck *et al.*^22^ failed to find any changes in ONSD, suggesting that the increases in ICP were small.

However, the generally low incidence of neurological complications after RAS makes it difficult to recommend more extensive screening procedures for intracranial hypertension. As described above, the steep Trendelenburg, when associated with pneumoperitoneum and mechanical ventilation, can cause a reduction in CO and cerebral hypoperfusion.^23^ The increase in *P*aCO_2_ due to CO_2_ insufflation can lead to a further increase in stasis of the brain blood flow and ICP due to pulmonary vasoconstriction and central venous hypertension. These changes could affect the onset of postoperative cognitive disorders including postoperative cognitive dysfunction (POCD) and postoperative delirium (POD).^3^

The relationship between impaired cerebrovascular status and postoperative neurological outcome in geriatric anaesthesia is currently under investigation. Nicolai Goettel *et al.*^24^ showed that the autoregulatory plateau in cerebral blood flow is shortened in patients under sevoflurane anaesthesia, without any effect of age. Moreover, impaired intra-operative cerebral autoregulation seems to be not predictive of early POCD in elderly patients after major noncardiac surgery.^25^ On the contrary, Chen *et al.* demonstrated that patients undergoing RALP may develop elevated ICP in addition to short term postoperative cognitive impairment. The combination of many factors, including anaesthesia, age, positioning and CO_2_ insufflation during RAS could predispose elderly patients to postoperative cognitive disorders, but this topic needs to be further investigated.

During the steep Trendelenburg position, the Bispectral Index (BIS) increases significantly. This could lead to a deepening of anaesthesia with dangerous repercussions on cognitive functions in the elderly.^26^ In elderly patients undergoing noncardiac surgery, maintaining a BIS value between 40 and 60 seems to protect against the development of postoperative disorders.^27^ During RAS, high thresholds (around 60) may be tolerated. We should also take into consideration that, even if more randomised studies are needed, BIS values <40–45 may increase long-term mortality (>1 year).^28^

## Variation in intra-ocular pressure

The increase in intra-ocular pressure (IOP) during steep Trendelenburg is a well known problem. Cases of injury to the optic nerve have been reported after RALP when patients are required to be placed in the steep Trendelenburg position. Prolonged head-down positions increase the risk of blindness in patients suffering from moderate or high-pressure glaucoma (frequent in the elderly). In some centres, cases of robotic prostatectomy or hysterectomy may be cancelled by the ophthalmologist because of advanced glaucoma.^29^ Recent literature classifies glaucoma according to the Hodapp–Parrish–Anderson Classification of Glaucoma Severity; a positive result is a mean deviation on Humphrey visual field test worse than −12 decibels.^30^

Strategies to avoid possible damage in patients with risk factors such as advanced age, hypertension, diabetes and macular degeneration have been investigated and include the following: reducing head-down to the lowest possible degree that permits good working condition for the surgeon; using dNMB; preferring total intravenous anaesthesia and/or dexmedetomidine instead of inhaled anaesthetics; using the modified Z position with horizontal head and shoulders. As for the position, the 25° Trendelenburg significantly attenuated a rise in IOP compared to 30° without increasing the effort of the surgeons. The increase in IOP seems to be affected also by an increase in *P*aCO_2_, leading to vasodilation in the choroid plexus.^31^ Yoo *et al.*^32^ showed that dNMB, defined as a posttetanic count of 1 to 2, results in a smaller increase in IOP when compared with moderate NMB (train of four count of 1 to 2). This finding can be explained by a greater relaxation of extra-ocular muscles, which helps aqueous humour drainage. The increase in IOP seems to be less with propofol than with sevoflurane during RAS in the head-down position.^33,34^ Moreover, a continuous infusion of dexmetomidine (0.4 μg kg^−1^ h^−1^ immediately after anaesthesia induction until the end of surgery) during propofol-based anaesthesia in patients undergoing RALP significantly reduced IOP.^35^Although IOP rapidly returns to baseline, the long-term effects of increased IOP on ocular structures remains unclear.

Hirooka *et al.*^36^ found that while the IOP increased during RALP, there were no ocular complications. Similarly, Mizumoto *et al.*^37^ found no detectable disorder of ocular structural variables analysed at three and six months after RALP. There were no significant differences in the ocular variables examined three months after the procedure in patients with a healthy ocular system. The practice of some groups prophylactically trying to lower the IOP during steep Trendelenburg remains questionable.

Further studies are needed to demonstrate that an increase in IOP correlates with adverse clinical outcomes, especially in patients with preexisting elevated IOP.

Previous studies raise an important concern about patients with a diseased ocular system as preexisting damage could be exacerbated during RAS, especially when steep Trendelenburg is required. An ophthalmological consultation for risk assessment could be safer in patients having a diseased ocular system and also optical coherence tomography to evaluate retinal thickness.^31,38^ No formal recommendations exist regarding preoperative vision screening or peri-operative medication for patients undergoing RAS with a known increased IOP. However, it has been suggested that patients with preexisting ophthalmological conditions should obtain a consultation before RAS.^38,39^

## Conclusions

RAS can be safely performed in the elderly, even if it requires a very close collaboration between anaesthesiologists and surgeons. All strategies to achieve the lowest possible pneumoperitoneum pressure and to reduce the time spent in extreme positions are encouraged in order to limit possible adverse effects that may impair postoperative outcomes.
